# Long-term Exposure to PM_10_ and NO_2_ in Association with Lung Volume and Airway Resistance in the MAAS Birth Cohort

**DOI:** 10.1289/ehp.1205961

**Published:** 2013-06-18

**Authors:** Anna Mölter, Raymond M. Agius, Frank de Vocht, Sarah Lindley, William Gerrard, Lesley Lowe, Danielle Belgrave, Adnan Custovic, Angela Simpson

**Affiliations:** 1 Centre for Occupational and Environmental Health, Health Sciences Group, School of Community-Based Medicine, Manchester Academic Health Sciences Centre, The University of Manchester, Manchester, United Kingdom; 2 Department of Geography, School of Environment, Education and Development, The University of Manchester, Manchester, United Kingdom; 3 Salford Lung Study, North West e-Health, Salford, United Kingdom; 4 Manchester Academic Health Science Centre, NIHR Translational Research Facility in Respiratory Medicine, University Hospital of South Manchester NHS Foundation Trust, Wythenshawe Hospital, Manchester, United Kingdom

## Abstract

Background: Findings from previous studies on the effects of air pollution exposure on lung function during childhood have been inconsistent. A common limitation has been the quality of exposure data used, and few studies have modeled exposure longitudinally throughout early life.

Objectives: We sought to study the long-term effects of exposure to particulate matter with an aerodynamic diameter ≤ 10 μm (PM_10_) and to nitrogen dioxide (NO_2_) on specific airway resistance (sR_aw_) and forced expiratory volume in 1 sec (FEV_1_) before and after bronchodilator treatment. Subjects were from the Manchester Asthma and Allergy Study (MAAS) birth cohort (*n* = 1,185).

Methods: Spirometry was performed during clinic visits at ages 3, 5, 8, and 11 years. Individual-level PM_10_ and NO_2_ exposures were estimated from birth to 11 years of age through a microenvironmental exposure model. Longitudinal and cross-sectional associations were estimated using generalized estimating equations and multivariable linear regression models.

Results: Lifetime exposure to PM_10_ and NO_2_ was associated with significantly less growth in FEV_1_ (percent predicted) over time, both before (–1.37%; 95% CI: –2.52, –0.23 for a 1-unit increase in PM_10_ and –0.83%; 95% CI: –1.39, –0.28 for a 1-unit increase in NO_2_) and after bronchodilator treatment (–3.59%; 95% CI: –5.36, –1.83 and –1.20%; 95% CI: –1.97, –0.43, respectively). We found no association between lifetime exposure and sR_aw_ over time. Cross-sectional analyses of detailed exposure estimates for the summer and winter before 11 years of age and lung function at 11 years indicated no significant associations.

Conclusions: Long-term PM_10_ and NO_2_ exposures were associated with small but statistically significant reductions in lung volume growth in children of elementary-school age.

Citation: Mölter A, Agius RM, de Vocht F, Lindley S, Gerrard W, Lowe L, Belgrave D, Custovic A, Simpson A. 2013. Long-term exposure to PM_10_ and NO_2_ in association with lung volume and airway resistance in the MAAS birth cohort. Environ Health Perspect 121:1232–1238. http://dx.doi.org/10.1289/ehp.1205961

## Introduction

Lung function is an important indicator of respiratory health and long-term survival (Hole et al. 1996). Unlike information collected through questionnaires, measured lung function is an objective health outcome that is not affected by recall or reporting bias. The respiratory tract is at risk from air pollution, because gaseous pollutants and small particles in the air are inhaled through the nose and mouth. Two air pollutants frequently studied are nitrogen dioxide (NO_2_) and particulate matter (PM). Both are derived from traffic related sources, but are also generated within the home—for example, by gas cookers and cigarette smoke. Both of these pollutants have been associated with respiratory and cardiovascular morbidity and mortality (Brunekreef and Holgate 2002). Several cross-sectional and longitudinal studies have been carried out on the association between NO_2_ and PM exposure and lung function in children. However, results of these studies have been disparate and conclusions inconsistent. Whereas some studies reported associations with lung volume only (Raizenne et al. 1996; Rojas-Martinez et al. 2007; Sugiri et al. 2006), others reported associations with expiratory flow only (Avol et al. 2001; Oftedal et al. 2008). Some studies reported associations with both lung volume and flow (Gauderman et al. 2000; Horak et al. 2002; Schwartz 1989), whereas others reported no associations at all (Dockery et al. 1989; Hirsch et al. 1999; Neas et al. 1991; Nicolai et al. 2003). In a recent review of studies on air pollution and lung function, Götschi et al. (2008) concluded that it was not possible to perform formal quantitative comparisons of findings because of the heterogeneity of study designs.

One limitation common to many previous studies lies in the assessment of exposure to air pollution. Most studies of the effects of air pollution on lung development in children have estimated associations with more recent air pollution exposure—the average concentration over the previous 12 months, rather than lifetime exposure or early-life exposure (Oftedal et al. 2008), and have estimated exposures based on measurements from central monitoring stations located near the child’s residence, without accounting for geographical factors (Hirsch et al. 1999; Nicolai et al. 2003; Oftedal et al. 2008), indoor as well as outdoor exposures, or time–activity patterns.

We have developed a novel microenvironmental exposure model (MEEM) (Mölter et al. 2012), which allows for spatial (indoor and outdoor microenvironments) and temporal variability in pollutant concentrations (Mölter et al. 2010a, 2010b) and incorporates children’s time–activity patterns to predict personal exposure. The performance of MEEM (for NO_2_) was evaluated previously through a personal monitoring study of 46 12- to 13-year-old schoolchildren in Manchester, United Kingdom (Mölter et al. 2012); we found good agreement between modeled and measured NO_2_ concentration (e.g., mean predictor error = –0.75; normalized mean bias factor = 0.04; normalized mean average error factor = 0.27; Spearman’s rank correlation = 0.31, *p* < 0.05) This performance evaluation also demonstrated that MEEM provided better estimates of exposure than central monitors or an outdoor air pollution model, which tended to overestimate personal exposure levels (Mölter et al. 2012).

The aim of the present study was to estimate the associations of modeled PM_10_ (particulate matter with an aerodynamic diameter ≤ 10 μm) and NO_2_ exposure with lung function in elementary-school children enrolled in a population-based birth cohort—the Manchester Asthma and Allergy Study (MAAS). Exposures and lung function were evaluated longitudinally throughout childhood. In addition, we applied a more detailed exposure model in a cross-sectional analysis of lung function measured at 11 years of age.

## Methods

*Study population.* The children studied were participants of MAAS, is an ongoing prospective birth cohort, which initially comprised 1,185 children of mothers who were recruited during pregnancy at two local hospitals between 1995 and 1997 (Simpson et al. 2001). Children attended review clinics at ages 3, 5, 8, and 11 years; the clinics included pulmonary function tests and skin prick tests for common inhalant and food allergens. In addition, parentally completed questionnaires were collected at each review (Custovic et al. 2002, 2004). MAAS received ethical approval by the Local Research Ethics Committee (SOU/00/258; SOU/00/259), and written informed consent was provided by the parents.

*Definition of outcomes: lung function.* All pulmonary function tests were performed by trained technicians at Wythenshawe Hospital, Manchester. The most informative test to measure lung function was selected for each age group (Beydon et al. 2007; Bisgaard and Klug 1995; Dab and Alexander 1976).

Specific airways resistance (sR_aw_) was measured at ages 3, 5, 8, and 11 years, using a constant volume whole-body plethysmograph (Masterscreen Body 4.3; Erich Jaeger GmbH, Würzburg, Germany) (Lowe et al. 2002; Nicolaou et al. 2008). High values of sR_aw_ indicate poor lung function. Forced expiratory volume in 1 sec (FEV_1_) was measured at ages 5, 8, and 11 years using a pneumotachograph-based spirometer (Erich Jaeger Gmbh). The protocol for measuring FEV_1_ was in accordance with American Thoracic Society guidelines (American Thoracic Society 1995). All children were asymptomatic at the time of testing, and β2-agonists were withheld for at least 4 hr before testing. The test was repeated at intervals of 30 sec until three technically acceptable traces were obtained, the highest two of which were within 5% of each other. The percent predicted FEV_1_ was calculated using reference equations developed by the Asthma UK Collaborative Initiative (Stanojevic et al. 2009). Postbronchodilator FEV_1_ was measured when the children were 5 and 11 years of age by repeating the FEV_1_ measurement 15 min after inhalation of 400 μg of albuterol. Results were analyzed as percent predicted FEV_1_.

*Definition of exposures: modeled PM_10_ and NO_2_ exposure.* The exposure estimates in this study are based on the concept of microenvironments (ME)—a defined space with a homogenous pollutant concentration (Ott 1982). MEs can represent spaces outdoors or indoors, and different methods can be used to estimate concentrations in different types of microenvironments. The microenvironmental models used in this study assumed that children spend the majority of their time in three types of MEs: home, school, and the journey between home and school.

Information on children’s home and school addresses from birth to 11 years of age was collected through a parental questionnaire, completed at the age 11 review. In this questionnaire parents were asked to list the dates and addresses for all homes the child had lived in and each school the child attended, the mode of transport between each home and respective schools. These data were entered into an SQL database (MS SQL2008R2; Microsoft, Redmond, WA, USA) to create a timeline for home and school addresses from birth to 11 years of age for each child. In addition, the shortest driving route between each home and school was estimated using the network analyst extension of ArcGIS9.2 (ESRI, Redlands, CA, USA).

**Figure 1:**
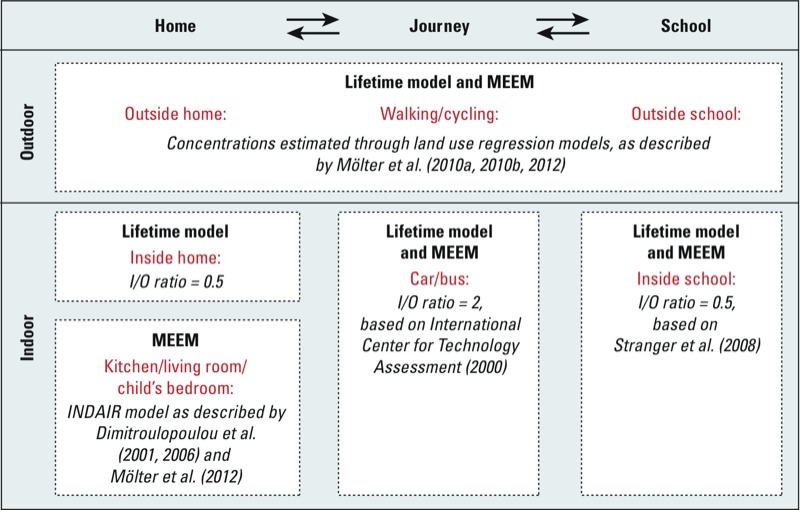
Outline of exposure assessment showing methods used to estimate concentrations in each microenvironment (with relevant references). The same methods were used at all time points except for the year before the age 11 review. A detailed indoor model could be used to estimate concentrations inside the kitchen, living room, and child’s bedroom. Abbreviations: I/O, Indoor to outdoor ratio; MEEM, micro­environmental exposure model.

Figure 1 summarizes the methods used to estimate NO_2_ and PM_10_ concentration in each ME. Concentrations for outdoor MEs (i.e., home outdoor ME, school outdoor ME, journey outdoor ME) were estimated using land use regression (LUR) models, as described in detail elsewhere (Mölter et al. 2010a, 2010b). In brief, LUR models were developed using estimated annual mean NO_2_ and PM_10_ concentrations at 208 locations derived from an air dispersion model. The final LUR models mainly comprised traffic-related predictor variables, such as vehicle counts on major roads, and had determination coefficients (*R*^2^) of 0.71. Performance evaluations using a set-aside data set (70 locations), and concentrations measured at automatic monitoring stations showed an acceptable level of agreement (*R*^2^ range, 0.33–0.86). To model children’s exposure from 1996 through 2008, the above LUR models were recalibrated to provide 13 annual models for PM_10_ and NO_2_, respectively (Mölter et al. 2010b): Data from the air dispersion model and the United Kingdom year adjustment calculator were used to estimate annual PM_10_ and NO_2_ concentrations from 1996 through 2008 at the 278 receptor sites described above. These concentrations were entered into regression analyses that included the same predictor variables used in the original LUR models. This resulted in individual models for each year; all models used the same predictor variables but generated different coefficients. A performance evaluation of these models against monitored data showed good agreement [*R*^2^ range, 0.35–0.97; root mean square error (RMSE) range, 1.8–8.3] (Mölter et al. 2010b).

Concentrations for journey indoor MEs (i.e., inside cars or buses) and school indoor MEs were estimated based on indoor to outdoor (I/O) ratios published in the literature (International Center For Technology Assessment 2000; Stranger et al. 2008). Concentrations in the Home indoor MEs were estimated using I/O ratios or a mass balance model (INDAIR), depending on the time period being modeled (Dimitroulopoulou et al. 2006). This resulted in two slightly different models: the MEEM and the lifetime models (Figure 1).

MEEM was used to estimate each child’s exposures during the summer and winter before the review visit at 11 years of age (Mölter et al. 2012). We modeled winter and summer exposures separately to capture variation in home indoor air concentrations because of seasonal differences in air exchange rates. In MEEM home kitchen ME, home living room ME, and home bedroom ME, concentrations were estimated individually using the INDAIR model, designed specifically to estimate indoor concentrations of NO_2_ and PM_10_ concentrations within residential buildings in the United Kingdom (Dimitroulopoulou et al. 2001, 2006).

A parent questionnaire administered at the child’s age 11 review was used to collect input parameters for the INDAIR model, such as room sizes, air exchange rates, and the presence of indoor sources of NO_2_ and PM_10_. The indoor sources included in the model were gas cooking and cigarette smoke, which are considered to be the main sources of NO_2_ and PM_10_ inside homes in the United Kingdom (Berry et al. 1996; Coward et al. 2001). In addition, the questionnaire collected time–activity data used to estimate the timing and duration of time in each ME. Therefore, MEEM provided spatially resolved time-weighted exposure estimates for each child.

We evaluated the performance of MEEM using a personal monitoring study of schoolchildren (12–13 years of age) attending a local secondary school in Manchester (Mölter et al. 2012). MEEM performed well when compared with NO_2_ concentrations measured with personal monitors (Ogawa passive samplers; Ogawa & Co. USA, Inc., Pompano Beach, FL, USA), with a mean prediction error of –0.75 μg/m^3^. A paired analysis of measured and predicted concentrations showed no significant difference between measured concentrations and MEEM estimates (Wilcoxon’s signed rank test: *z* = –0.05, *p* = 0.96).

Input parameters for the INDAIR model were available for the current (at 11 years of age) home of each child, but most children had changed residence at least once since birth. Therefore, we used a simplified lifetime model to estimate the average PM_10_ and NO_2_ exposure of each child for each month from birth to 11 years. In contrast with MEEM, the lifetime model used an I/O ratio to calculate exposure inside the home, instead of using the INDAIR model, and it assumed that all children were in the school indoor ME from 0900 to 1500 hours. However, as for MEEM, outdoor ME exposures (i.e., home outdoor ME, school outdoor ME, journey outdoor ME) were estimated using LUR models, and journey indoor MEs (i.e., inside cars or buses) and school indoor MEs were estimated based on I/O ratios.

*Definition of potential confounders.* Potential confounding variables and covariates were identified based on previous research within MAAS and previous publications (Lowe et al. 2002, 2004; Nicolaou et al. 2008; Oftedal et al. 2008) and included sex, age, ethnicity, older siblings, sensitization, asthma or current wheeze, family history of asthma, parental smoking, parental atopy, child care attendance during the first 2 years of life, hospitalization during the first 2 years of life, presence of a gas cooker in the home, presence of a dog or cat in the home, visible signs of dampness or mold in the home, body height, body weight, body mass index, maternal age at birth, gestational age, duration of breastfeeding, Tanner stage (age 11 years only), and socioeconomic status (paternal income). In addition, average PM_10_ and NO_2_ concentrations over 3 days before the child’s review visit were collected from four (for PM_10_) or five (for NO_2_) urban background monitoring stations across the Greater Manchester area (Oftedal et al. 2008).

We classified children as having current wheeze based on a positive response to the question “Has your child had wheezing or whistling in the chest in the last 12 months?” and classified them as having asthma based on positive answers to at least two of the following three variables: doctor diagnosis of asthma ever; current wheeze; asthma medication during the previous 12 months, consistent with the GA^2^LEN (Global Allergy and Asthma European Network) definition of asthma (Carlsen et al. 2006; Håland et al. 2006). At each review, potential allergic sensitization to common inhalant and food allergens was determined through skin prick tests for inhalant allergens (mites, cat, dog, mold, grass pollen, and tree pollen) and food allergens (milk, egg, and peanut). All allergens were tested at each review except for tree pollen and peanut allergens, which were tested at the age 8 and age 11 reviews only. Children were classified as having atopy, if they had at least one positive skin prick test (defined as a mean wheal diameter 3 mm greater than the negative control). Parental atopy was also established through skin prick tests, which were carried out during the recruitment stage.

*Statistical analysis.* All analyses were carried out with SPSS 16.0 (IBM SPSS, Chicago, IL, USA). Before all analyses, sR_aw_ was ln-transformed because it follows a log-normal distribution. FEV_1_ and postbronchodilator FEV_1_ were not transformed because these variables were normally distributed. Multivariable linear regression was used to cross-sectionally estimate associations of PM_10_ and NO_2_ exposure during the summer and winter before children were 11 years of age (estimated by MEEM), with sR_aw_ and FEV_1_ at 11 years. All potential confounders were entered individually into bivariate models with the exposure and outcome variables, and potential confounders that were significant predictors of the outcome (*p* < 0.05) were evaluated using multivariate stepwise analyses that retained only covariates that significantly predicted the outcome, or that were retained *a priori* (age and sex in all sR_aw_ models, Tanner stage for all models of outcomes at age 11). Models of FEV_1_ outcomes were not adjusted for age, sex, and body height, because these factors were used to calculate the percent predicted values. Models of MEEM exposures at 11 years of age were not adjusted for cigarette smoking because information on smoking was already included in the INDAIR model.

We analyzed the association between lifetime exposure and the development of lung function using generalized estimating equations to account for the within-subject correlation of repeated measures, with the same covariates included in the cross-sectional models. Monthly exposures were averaged into the following time windows: for sR_aw_, 0–3, 3–5, 5–8, and 5–11 years of age; for FEV_1_, 0–5, 5–8, and 8–11 years of age; for FEV_1_ after bronchodilator treatment, 0–5 and 5–11 years of age. For completeness, exposure estimates from the lifetime exposure model were also analyzed cross-sectionally against lung function at 3, 5, 8, and 11 years of age. For these analyses the monthly exposure estimates were averaged into the following time windows: first year of life (0–1), birth to review ages (0–3, 0–5, 0–8, 0–11 years), 1 calendar year before reviews (2–3, 4–5, 7–8, 10–11 years). The level for statistical significance was set at *p* < 0.05.

## Results

**Figure 2:**
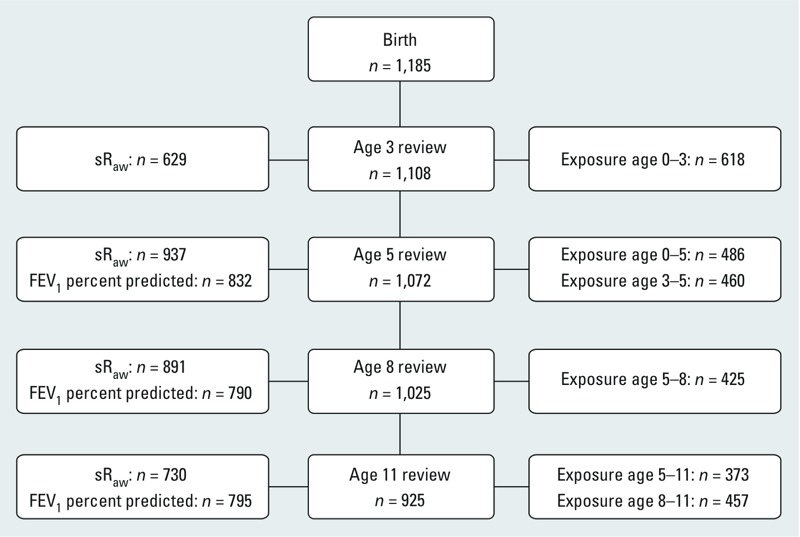
Flow diagram of MAAS cohort showing participation rates at each review, the number of lung function measurements collected, and the number of exposure estimates available.

*Participants and descriptive data.* Participant flow with numbers of individuals at each stage of the study, the number of lung function measurements collected and the number of exposure estimates available is shown in Figure 2. Descriptive statistics of the study population and the covariates included in the final models are presented in Table 1; descriptive statistics of potential confounders not included in the final models are shown in Supplemental Material, Table S1. As expected, the prevalence of atopy increased from 3 to 11 years of age, whereas the prevalence of asthma or current wheeze remained fairly constant during this time period. A complete data set of FEV_1_, pollutant exposures, and covariates at two or more reviews was available for 342 children (Table 1). Children included in the longitudinal analysis of the effect of PM_10_ and NO_2_ exposure on the change in FEV_1_ were more likely to be female and were less likely to have asthma or wheeze in early life. By 8 years of age, there were no differences in asthma/wheeze between children with full sets of longitudinal data and those without. Table 2 summarizes the lung function measurements at each age. The mean FEV_1_ increased from 1.05 L at 5 years to 2.30 L at 11 years, resembling typical values for Caucasian children of these ages (Stanojevic et al. 2009).

**Table 1:** Description of study population.

Variable	MAAS cohort at birth		Children with longitudinal FEV_1_ and longitudinal exposure data		*p-*Value^*c*^
	*N*^*a*^	*n*^*b*^ (%) or mean ± SD	*N*^*a*^	*n*^*b*^ (%) or mean ± SD	
Female sex	1,185	543 (45.8)	342	173 (50.6)	0.036
Family history of asthma	1,185	441 (37.2)	342	125 (36.5)	0.763
Child is atopic^*d*^					
Age 3	983	225 (22.9)	306	72 (23.5)	0.748
Age 5	963	294 (30.5)	334	94 (28.1)	0.241
Age 8	927	314 (33.9)	330	100 (30.3)	0.088
Age 11	784	281 (35.8)	332	116 (34.9)	0.652
Child has asthma or current wheeze
Age 3	1,097	296 (27.0)	330	71 (21.5)	0.007
Age 5	1,071	297 (27.7)	341	75 (22.0)	0.004
Age 8	1,023	217 (21.2)	341	65 (19.1)	0.234
Age 11	925	214 (23.1)	341	78 (22.9)	0.886
Hospitalization during first 2 years of life for lower respiratory tract infection	1,185	109 (9.2)	342	34 (9.9)	0.573
Gas cooker in the home
Age 1	1,028	801 (77.9)	341	270 (79.2)	0.492
Age 8	1,029	819 (79.6)	342	270 (78.9)	0.717
Age 11	930	727 (78.2)	342	267 (78.1)	0.954
Age at follow-up (years)
Age 3	1,081	3.0 ± 0.1	326	3.0 ± 0.0	0.208
Age 5	1,044	5.0 ± 0.1	340	5.0 ± 0.1	0.008
Age 8	976	8.0 ± 0.2	339	8.0 ± 0.1	0.084
Age 11	813	11.4 ± 0.5	341	11.4 ± 0.5	0.876
Body mass index (kg/m²)
Age 3	1,044	16.7 ± 1.4	321	16.7 ± 1.5	0.914
Age 5	1,017	16.3 ± 1.6	339	16.4 ± 1.7	0.776
Age 8	923	17.1 ± 2.4	333	17.1 ± 2.6	0.643
Age 11	816	19.1 ± 3.4	341	19.2 ± 3.4	0.885
Short-term PM_10_ (μg/m^3^) 3-day average before review visit
Age 3	1,081	21.6 ± 7.7	326	21.0 ± 6.9	0.186
Age 5	1,044	21.5 ± 7.2	340	21.6 ± 7.2	0.910
Age 8	976	20.8 ± 6.2	339	21.0 ± 6.0	0.660
Age 11	820	19.6 ± 9.2	337	19.7 ± 9.0	0.895
Mean Tanner stage	763	2.1 ± 0.9	317	2.1 ± 0.9	0.648
^***a***^Total number of children. ^***b***^Number of positive children. ^***c***^*p*-Value of chi-square test or Student’s *t*-test comparing children with longitudinal FEV_1_ and exposure data against all children in the MAAS cohort at birth. ^***d***^Determined through skin prick test, mean wheal diameter 3 mm greater than negative control for at least 1 of 9 allergens tested.					

**Table 2:** Summary of lung function measures at each review (mean ± SD).

Lung function measure	Age 3	Age 5	Age 8	Age 11
sR_aw_ (kPa/sec)^*a*^	1.10 (1.23)	1.17 (1.21)	1.22 (1.23)	1.26 (1.29)
FEV_1_ (L)		1.05 ± 0.16	1.59 ± 0.25	2.30 ± 0.40
Predicted FEV_1_ (L)		1.03 ± 0.27	1.60 ± 0.17	2.34 ± 0.29
FEV_1_ (% predicted)		96.4 ± 12.7	99.0 ± 11.8	98.5 ± 11.7
FEV_1_ postbronchodilator (% predicted)		104.9 ± 11.3		103.8 ± 11.5
^***a***^Geometric mean (GSD).				

*Exposure to pollutants.* Figures S1 and S2 (Supplemental Material) describe the distribution of the exposure estimates by pollutant and exposure time window. MEEM predicted higher PM_10_ and NO_2_ exposures during the winter than during the summer (see Supplemental Material, Figures S1 and S2), and it predicted a wider range of exposures than the lifetime model. The lifetime exposure estimates decreased from 0–1 to 10–11 years of age (see Supplemental Material, Figures S1 and S2), which most likely reflects the general decrease of PM_10_ and NO_2_ levels in the Greater Manchester area from 1996 to 2008 (Department for Environment, Food and Rural Affairs 2009). PM_10_ and NO_2_ exposures were moderately to strongly correlated in all exposure time windows (Pearson’s *r* = 0.59–0.89).

*Association between exposure to pollutants and sR_aw_.* The results of the cross-sectional analyses conducted at 3–11 years of age are shown in Supplemental Material, Table S2. Table S2 indicates a significant negative association between PM_10_ exposure during early life and sR_aw_ at 3 and 5 years. However, all other analyses showed no statistically significant associations. Furthermore, at 11 years there was no association between PM_10_ and NO_2_ exposure (MEEM) during the summer or winter and sR_aw_ (Table 3), and there was no association between lifetime exposure and longitudinal sR_aw_.

**Table 3:** Results of longitudinal analyses (GEE) of longitudinal PM_10_ and NO_2_ exposure (based on the lifetime model) and lung function and cross-sectional analy­ses (multivariable linear regression) of PM_10_ and NO_2_ exposure at 10–11 years of age (based on the lifetime model or MEEM) and lung function at 11 years of age.

Exposure metric/lung function metric	Longitudinal exposure and lung function			Exposure at age 10–11 (lifetime model) and lung function at age 11			Winter exposure before age 11 review (MEEM) and lung function at age 11			Summer exposure before age 11 review (MEEM) and lung function at age 11		
	β^*a*^ (95% CI)	*p-*Value	*n*^*b*^	β^*a*^ (95% CI)	*p-*Value	*n*^*b*^	β^*a*^ (95% CI)	*p-*Value	*n*^*b*^	β^a^ (95% CI)	*p-*Value	*n*^*b*^
PM_10_ (μg/m^3^)
Ln sR_aw_ (kPa/sec)^*c*^	0.009 (–0.027, 0.010)	0.37	453	–0.007 (–0.054, 0.040)	0.77	352	–0.001 (–0.011, 0.008)	0.78	315	0.001 (–0.008, 0.009)	0.90	298
FEV_1_ (% predicted)^*d*^	–1.37 (–2.52, –0.23)	0.019	342	–1.13 (–3.36, 1.09)	0.32	373	–0.20 (–0.65, 0.26)	0.39	334	0.07 (–0.33, 0.47)	0.73	317
FEV_1_ after bronchodilator treatment (% predicted)^*d*^	–3.59 (–5.36, –1.83)	< 0.001	176	–1.71 (–3.94, 0.53)	0.13	366	–0.14 (–0.61, 0.34)	0.57	327	0.15 (–0.27, 0.57)	0.48	310
NO_2_ (μg/m^3^)
Ln sR_aw_ (kPa/sec)^*c*^	–0.007 (–0.016, 0.003)	0.16	453	0.002 (–0.020, 0.023)	0.88	352	0.001 (–0.004, 0.007)	0.64	315	–0.001 (–0.006, 0.004)	0.57	298
FEV_1_ (% predicted)^*d*^	–0.83 (–1.39, –0.28)	0.003	342	–0.83 (–1.79, 0.14)	0.093	373	–0.10 (–0.36, 0.17)	0.47	334	0.05 (–0.18, 0.29)	0.66	317
FEV_1_ after bronchodilator treatment (% predicted)^*d*^	–1.20 (–1.97, –0.43)	0.002	176	–1.00 (–1.96, –0.03)	0.043	366	–0.01 (–0.29, 0.27)	0.93	327	0.08 (–0.17, 0.32)	0.53	310
GEE, generalized estimating equation.^***a***^β coefficient per 1-μg/m^3^ increase in exposure. ^***b***^Number of children included in analysis. ^***c***^Adjusted for age, sex, concurrent body mass index, concurrent atopy, concurrent asthma or wheeze, family history of asthma, hospitalization during first two years of life for lower respiratory tract infection, average 3-day background PM_10_ concentration prior to sR_aw_ ­measurement, mean Tanner stage. ^***d***^Adjusted for age (only in GEE), concurrent atopy, concurrent asthma or wheeze, hospitalization during first two years of life for lower respiratory tract infection, gas cooker in home, mean Tanner stage.												

*Association between exposure to pollutants and FEV_1_.* In the cross-sectional analysis at 11 years of age, there was no association between PM_10_ and NO_2_ exposure (MEEM) during the summer or winter and FEV_1_ percent predicted (Table 3). In contrast, the longitudinal model of lifetime exposure to pollutants and longitudinal measures of FEV_1_ revealed a significant association between exposure to pollutants and the change in this measure of lung function during childhood. PM_10_ and NO_2_ exposures were associated with poorer lung function over time [PM_10_: β = –1.37 (95% CI: –2.52, –0.23); NO_2_: β = –0.83 (95% CI: –1.39, –0.28)]. Based on the average predicted FEV_1_ within MAAS at 5, 8, and 11 years of 1.65 L (Table 2), the model estimated that for each unit increase (1 μg/m^3^) in PM_10_ exposure, the growth in FEV_1_ from 5 to 11 years was 23 mL smaller; and for each unit increase (1 μg/m^3^) of NO_2_ exposure, the growth in FEV_1_ was 14 mL smaller [ΔFEV_1_ = β / 100 × 1.65 × 1,000]. Results of cross-sectional analyses conducted at other time points are shown in Supplemental Material, Table S3; we observed no statistically significant association between PM_10_ or NO_2_ exposure windows and FEV_1_ in cross-sectional analyses.

*Association between exposure to pollutants and postbronchodilator FEV_1_.* At 11 years of age, there was no association between PM_10_ or NO_2_ exposure (MEEM) during the summer or winter and postbronchodilator FEV_1_ percent predicted (Table 3). However, there was a significant negative association between postbronchodilator FEV_1_ and the annual average NO_2_ exposure from 10 to 11 years of age estimated by the lifetime model (β = –1.00; 95% CI: –1.96, –0.03, *p* = 0.043). In the longitudinal models, we observed a significant negative association between postbronchodilator FEV_1_ and PM_10_ and NO_2_ exposure over time [PM_10_: β = –3.59 (95% CI: –5.36, –1.83); NO_2_: β = –1.20 (95% CI: –1.97, –0.43)]. Based on the average predicted FEV_1_ of 1.65 L, these would be equivalent to a growth deficit in post bronchodilator FEV_1_ of 59 mL from 5 to 11 years of age per unit increase in PM_10_, and a growth deficit of 20 mL from 5 to 11 years per unit increase in NO_2_. For completeness results of cross-sectional analyses conducted at other time points are shown in Supplemental Material, Table S4. Table S4 shows significant negative associations between postbronchodilator FEV_1_ and early-life PM_10_ (β_Age 0–1_ = –3.00; 95% CI: –5.29, –0.71; β_Age 0–5_ = –4.70; 95% CI: –7.85, –1.55) and NO_2_ exposures (β_Age 0–1_ = –0.91; 95% CI: –1.77, –0.05).

## Discussion

To our knowledge, this is the first study to estimate the effect of modeled individual lifetime exposure to PM_10_ and NO_2_, from birth through elementary school, on the development of lung function measured throughout childhood. With both exposure and lung function modeled longitudinally, our results indicated a small but statistically significant impairment in growth of FEV_1_ with an increase in exposure to air pollutants. We estimated the size of this effect to be a loss of 23 mL in the growth in FEV_1_ from 5 to 11 years of age per unit increase in PM_10_ (~ 3.8 mL/year), and 14 mL per unit increase of NO_2_ exposure (~ 2.3 mL/year). In addition, we observed significant associations of PM_10_ and NO_2_ exposures with postbronchodilator FEV_1_. In cross-sectional analyses, using a detailed assessment of summer and winter pollutant exposure at 11 years, we found no associations between air pollution and contemporaneous measures of lung function.

One of the strengths of this study was the use of the comprehensive validated MEEM model to estimate exposures for cross-sectional analyses of outcomes at 11 years of age. This model provided weighted estimates of exposure based on time–activity patterns and NO_2_ and PM_10_ models with a high spatiotemporal resolution. Ideally, we would have used MEEM to estimate lifetime exposure of each child. However, MEEM requires detailed descriptions of the house design that were not available longitudinally for the approximately 50% of children who had moved house from their original home during follow-up. Therefore we used the lifetime model—a slightly simplified version of MEEM that did not require detailed knowledge of the home environment to estimate exposures on a monthly basis from birth to 11 years for longitudinal analyses. The ranges of exposures estimated by MEEM (9.7–28.0 μg/m^3^ and 6.5–38.1 μg/m^3^ for PM_10_ during the previous summer and winter, respectively; and 9.5–43.0 μg/m^3^ and 10.3–47.2 μg/m^3^ for NO_2_, respectively) were greater than the corresponding estimates from the lifetime model at 10–11 years (PM_10_: 8.8–14.0 μg/m^3^; NO_2_: 10.8–23.7 μg/m^3^). Differences between estimates from each model reflect the different time periods used for averaging (3-month averages during summer and winter for MEEM, 12-month averages at 10–11 years of age for the lifetime model) and the use of the INDAIR model to estimate indoor exposures for MEEM, which captures peaks in exposure due to gas cooking and cigarette smoking, as well as very low exposures due to low air exchange rates. However, the lifetime model also improves over previously used exposure assessment methods by providing retrospective estimates of monthly exposures that can be aggregated into different exposure time windows for longitudinal and cross-sectional analyses. Furthermore, using home and school address histories, we modeled exposure at an individual level, rather than a community level, thereby reducing the potential for exposure misclassification.

Because of the strong correlation between NO_2_ and PM_10_ exposures in our study, we used single- rather than two-pollutant models. Many previous cohort studies of air pollution have included cigarette smoking and socioeconomic status as confounders in their analysis (Brunst et al. 2012; Li et al. 2000; Stocks and Dezateux 2003). Although it is likely that parental smoking and socioeconomic status affect lung function in children, we did not include them in our final model because they were not significant predictors of the outcomes, and we therefore assumed that they did not confound associations with air pollution exposures in our study. However, we cannot rule out residual confounding by these or other exposures. In addition, we acknowledge that our estimates of PM_10_ exposures do not necessarily represent the size fraction of particulate matter that is most damaging and that further studies of associations with fine or ultrafine particles are needed to address this.

Another strength of this study was its setting in the context of a population-based birth cohort with repeated measurements of lung function—an objective outcome that is not affected by recall or reporting bias—at four ages. Assessment of sR_aw_ enabled measurement of lung function from a young age (3 years). Assessing bronchodilator responses is a common diagnostic tool to test for reversible airway obstruction that can also be used to estimate the maximum achievable expiratory volume of a child. The results of our longitudinal analyses suggest an average annual growth deficit of 9.8 mL/year and 3.3 mL/year in the maximum achievable expiratory volume with each unit increase in PM_10_ and NO_2_ exposure.

A limitation of this study was the relatively small sample sizes for some of the analyses, mostly due to missing exposure data. Exposure data were missing for children who moved outside the Greater Manchester area and for children with incomplete information on home and school addresses. However, the loss in precision due to sample size limitations may be partly offset by the use of detailed individual-level estimates of longitudinal exposures.

Most published studies have investigated the association between pollutant exposure and FEV_1_ cross-sectionally—at a single time point only. Some of these studies also reported that PM_10_ or NO_2_ exposures were associated with decreases in mean FEV_1_, but not at a statistically significant level (Avol et al. 2001; Dockery et al. 1989; Oftedal et al. 2008). However, other studies have reported significant negative associations between air pollution exposure and FEV_1_ (Gauderman et al. 2000, 2004; Horak et al. 2002; Peters et al. 1999; Rojas-Martinez et al. 2007), but often only in subgroups of children [e.g., only in girls (Peters et al. 1999), only in one age group (Gauderman et al. 2004), or only during one season (Horak et al. 2002)].

Few studies have estimated the longitudinal effects of pollutants on the growth in lung function (Table 4). The Children’s Health Study was set in 12 communities of Southern California (USA), with a broad range of pollutant exposures (Gauderman et al. 2000, 2004). After 4 years of follow-up from 10 years of age, increasing community exposure to PM_10_ was associated with a reduced adjusted mean FEV_1_ growth rate, with those in the most polluted community having an estimated cumulative reduction in FEV_1_ of 3.4% over 4 years compared with those in the least polluted communities (Gauderman et al. 2000). After 8 years of follow-up, this association with PM_10_ was no longer statistically significant, although a much higher proportion of the children who lived in high-PM_10_ communities had a FEV_1_ < 80% predicted. By the time children were 18 years of age, the average FEV_1_ in the community with the highest NO_2_ exposure was about 100 mL lower than that seen in the community with the lowest exposure (Gauderman et al. 2004). In a population of 975 8-year-old Austrian children who were followed for 3 years, significant negative associations with lung function growth were reported for winter NO_2_ and summer PM_10,_ even though higher concentrations of PM_10_ were present during the winter (Horak et al. 2002). A 3-year study of 3,170 children living in Mexico City, which has comparatively high pollution levels, reported statistically significant negative associations of both PM_10_ and NO_2_ with growth in FEV_1_ (Rojas-Martinez et al. 2007). Specifically, the authors estimated that an interquartile range (IQR) increase in PM_10_ (36.4 μg/m^3^) was associated with a mean annual deficit in FEV_1_ of 29 mL in girls and 27 mL in boys. Similarly, they estimated that an IQR increase in NO_2_ (12.0 ppb) was associated with a mean annual deficit of 32 mL in girls and 26 mL in boys. When estimates are scaled to the same exposure increment and time period (Table 4), it is apparent that past and present longitudinal studies have estimated a very broad range of effect sizes on lung function growth.

**Table 4:** Comparison of average deficit in lung growth with findings from previously published population-based studies.

Reference, country	Exposure assigned at	Study duration	Range of exposures (μg/m^3^)		Average deficit in lung growth (mL/year) associated with 1-μg/m^3^ increase in exposure^*a*^	
			PM_10_	NO_2_	PM_10_	NO_2_
Gauderman et al. 2000, 2004, USA	Community level	Age 10–14	20–65	10–70	0.20	0.19
Horak et al. 2002, Austria	Community level	Age 8–11	9–31	2–35	8.4	9.5
Rojas-Martinez et al. 2007, Mexico	Community level	Age 8–11	53–96	54–74	0.80 (girls), 0.74 (boys)	1.4 (girls), 1.1 (boys)
Present study, United Kingdom	Individual level	Birth–age 11	10–16	15–28	3.8	2.3
^***a***^Calculated based on published figures, assuming a linear relationship between exposure and lung function.						

Having found a longitudinal association during childhood, we find it interesting to speculate at which time point exposure to pollutants may be most damaging to lung function. The cross-sectional analysis of the detailed NO_2_ and PM_10_ exposure estimates derived from MEEM showed no association between exposure and lung function at 11 years of age. However, for postbronchodilator FEV_1_ the cross-sectional analyses indicate that early exposures are associated with poorer lung function (see Supplemental Material, Table S4), but this association was not as evident for FEV_1_ percent predicted (see Supplemental Material, Table S3). Previous research has suggested that lung development during infancy is particularly susceptible to environmental toxins and that exposure can result in irreversible lung damage (Dietert et al. 2000; Plopper and Fanucchi 2000). In the Children’s Health Study, no significant associations of pollutant exposures were reported for older children (recruited at 13 and 15 years of age) who were also followed longitudinally (Gauderman et al. 2000). However, most epidemiological studies on children’s lung function have assessed only present air pollution exposure (Götschi et al. 2008), and very little work has been done on early-life exposure (Oftedal et al. 2008). The results of the present study support the hypothesis that early life exposures may affect lung development in later life.

We found evidence of an impairment in lung function growth at apparently lower exposure levels than those of previous longitudinal studies of air pollution exposure and lung function in children (Avol et al. 2001; Gauderman et al. 2004; Rojas-Martinez et al. 2007). However, exposure estimates in previous studies are not directly comparable with exposure estimates used in our study, because they were based on levels measured at centrally located outdoor pollution monitors. In contrast, our estimates accounted for both indoor and outdoor exposures, because children living in urban areas in industrialized countries spend most of their time indoors (Infante-Rivard 1993). Our previous work on MEEM has shown that a model allowing for indoor and outdoor exposure provides a better estimate of personal exposure than methods based solely on outdoor air pollution, which tended to overestimate personal exposure (Mölter et al. 2012). Therefore, it is possible that exposure levels assigned to children in previous studies based on outdoor monitors overestimated their true personal exposures. Nonetheless, the maximum outdoor concentrations of 70–80 μg/m^3^ NO_2_ and 60–90 μg/m^3^ PM_10_ found in previous studies in Mexico (Rojas-Martinez et al. 2007) and the United States (Avol et al. 2001; Gauderman et al. 2004) do exceed the current regulatory limits for annual mean concentrations in the United Kingdom (NO_2_ = 40 μg/m^3^; PM_10_ = 40 μg/m^3^) and are higher than concentrations typically measured at urban background monitoring stations in Manchester (Mölter et al. 2010a, 2010b).

## Conclusions

Our findings suggest that lifetime exposure to PM_10_ and NO_2_ may be associated with reduced growth in FEV_1_ in children. Although the observed reductions in FEV_1_ growth were small, and therefore may have little impact on healthy individuals, they could have implications for individuals with chronic respiratory disease, particularly obstructive lung diseases, or in children who go on to smoke cigarettes. Future follow-up will provide further insight on whether reductions in FEV_1_ growth associated with air pollution persist into adulthood or disappear during adolescence.

## Supplemental Material

(979 KB) PDF
