# Supplementary material for: Long-term Exposure to PM10 and NO2 in Association with Lung Volume and Airway Resistance in the MAAS Birth Cohort
Source: Environ Health Perspect. 2013 Jun 18;121(10):1232–8. doi: 10.1289/ehp.1205961 (PMC3801452; doi:10.1289/ehp.1205961)
Supplement: (979 KB) PDF [file ehp.1205961.s001.pdf]

Environmental Health Perspectives doi:10.1289/ehp.1205961

SUPPLEMENTAL MATERIAL

Long-term Exposure to PM_{10} and NO_2 in Association with Lung Volume and Airway Resistance in the MAAS Birth Cohort

Anna Mölter, Raymond M. Agius, Frank de Vocht, Sarah Lindley, William Gerrard, Lesley Lowe, Danielle Belgrave, Adnan Custovic, and Angela Simpson

CONTENTS

List of Tables

Table S1: Descriptive statistics of potential confounders not included in the final models......2
Table S2 : Association between lifetime PM_{10} and NO_2 exposure (in µg/m³) and specific airway resistance at age 3, 5, 8 and 11 years[a]......6
Table S3: Association between lifetime PM_{10} and NO_2 exposure (in µg/m³) and FEV_1 (% predicted) at age 5, 8 and 11 years[a]......7
Table S4: Association between lifetime PM_{10} and NO_2 exposure (in µg/m³) and FEV_1 post bronchodilator (% predicted) at age 5 and 11[a]......8

List of Figures

Figure S1: Boxplots of estimated PM_{10} exposures (µg/m³) during the first year of life, one year prior to the review visit [derived from lifetime exposure model] and summer and winter before the age 11 review visit [derived from MEEM] (indicating the median, interquartile range, minimum, maximum and outliers)......4
Figure S2: Boxplots of estimated NO_2 exposures (µg/m³) during the first year of life, one year prior to the review visit [derived from lifetime exposure model] and summer and winter before the age 11 review visit [derived from MEEM] (indicating the median, interquartile range, minimum, maximum and outliers)......5

Table S1: Descriptive statistics of potential confounders not included in the final models

Variable	MAAS cohort at birth			Children with longitudinal FEV_1 and longitudinal exposure data			
	N	n or mean	(% or ±SD)	N	n or mean	(% or ±SD)	p[a]
Caucasian ethnicity	1115	1059	(95.0)	340	328	(96.5)	0.131
Older siblings	1073	561	(52.3)	341	179	(52.5)	0.925
Mother is atopic[b]	1147	683	(59.5)	339	203	(59.9)	0.881
Father is atopic[b]	1138	717	(63.0)	339	216	(63.7)	0.746
Maternal smoking during pregnancy	1025	119	(11.6)	341	29	(8.5)	0.028
Any smoker living in the home							
first year of life	1024	438	(42.8)	341	143	(41.9)	0.702
age 3	1080	337	(31.2)	329	90	(27.4)	0.071
age 5	1094	373	(34.1)	340	102	(30.0)	0.055
age 8	999	366	(36.6)	338	122	(36.1)	0.799
age 11	896	232	(25.9)	335	80	(23.9)	0.288
Paternal income at birth							
<£10000	1078	171	(15.9)	324	47	(14.5)	0.014
£10000-£20000	1078	406	(37.7)	324	125	(38.6)	
£20000-£30000	1078	297	(27.6)	324	106	(32.7)	
>£30000	1078	204	(17.2)	324	46	(14.2)	
Day-care attendance during the first two years of life	1087	741	(68.2)	329	224	(68.1)	0.969
Dampness or mould in the home							
age 1	1030	177	(17.2)	342	59	(17.3)	0.968
age 8	1030	149	(14.5)	342	56	(16.4)	0.220
age 11	930	143	(15.4)	342	50	(14.6)	0.626
Dog in the home							
first year of life	1028	174	(16.9)	342	52	(15.2)	0.299
age 3	1070	150	(14.0)	327	39	(11.9)	0.191
age 5	1090	168	(15.4)	340	39	(11.5)	0.015
age 8	1028	198	(19.3)	342	57	(16.7)	0.136
age 11	930	239	(25.7)	342	81	(23.7)	0.284
Cat in the home							
first year of life	1027	205	(20.0)	342	64	(18.7)	0.480
age 3	1068	208	(19.5)	327	51	(15.6)	0.033

Variable	MAAS cohort at birth			Children with longitudinal FEV_1 and longitudinal exposure data			
	N	n or mean	(% or ±SD)	N	n or mean	(% or ±SD)	p[a]
age 5	1093	216	(19.8)	340	51	(15.0)	0.008
age 8	1028	217	(21.1)	342	62	(18.1)	0.098
age 11	930	228	(24.5)	342	78	(22.8)	0.355
Height (cm)							
Girls							
age 3	483	95.0	(3.6)	161	95.0	(3.6)	0.945
age 5	465	109.5	(4.4)	171	109.8	(4.6)	0.405
age 8	447	127.6	(5.4)	172	128.1	(5.7)	0.295
age 11	386	148.9	(7.5)	173	149.6	(7.9)	0.344
Boys							
age 3	562	96.0	(3.7)	160	96.1	(3.7)	0.858
age 5	553	110.4	(4.5)	168	110.3	(4.6)	0.731
age 8	528	128.6	(5.7)	167	128.4	(5.5)	0.712
age 11	433	147.6	(7.8)	168	147.6	(7.1)	0.989
Weight (kg)							
age 3	1051	15.3	(1.9)	326	15.3	(2.1)	0.880
age 5	1019	19.9	(2.9)	339	19.9	(3.1)	0.735
age 8	976	28.2	(5.6)	339	28.3	(6.0)	0.606
age 11	816	42.4	(10.4)	340	42.7	(10.2)	0.707
Birthweight (g)	1132	3462	(510)	330	3496	(519)	0.302
Gestational age (wks)	1072	39.9	(1.6)	314	39.9	(1.6)	0.831
Maternal age at birth (yrs)	1115	30.4	(4.8)	329	31	(4.6)	0.030
Duration of breastfeeding (wks)	1115	15.5	(20.0)	329	19.4	(19.6)	0.002
Short term NO_2 ($\mu g/m^3$) 3 day average before review visit							
age 3	1081	30.6	(11.6)	326	29.9	(11.3)	0.324
age 5	1044	30.2	(11.9)	340	30.4	(12.1)	0.797
age 8	976	28.6	(10.0)	339	29.3	(10.2)	0.243
age 11	805	27.8	(13.7)	337	27.8	(13.4)	0.959

[a] p value of chi-square test or t-test comparing children with longitudinal FEV_1 and exposure data against all children in MAAS cohort at birth

[b] determined through skin prick test, mean wheal diameter 3mm greater than negative control for at least 1 out of 9 allergens tested

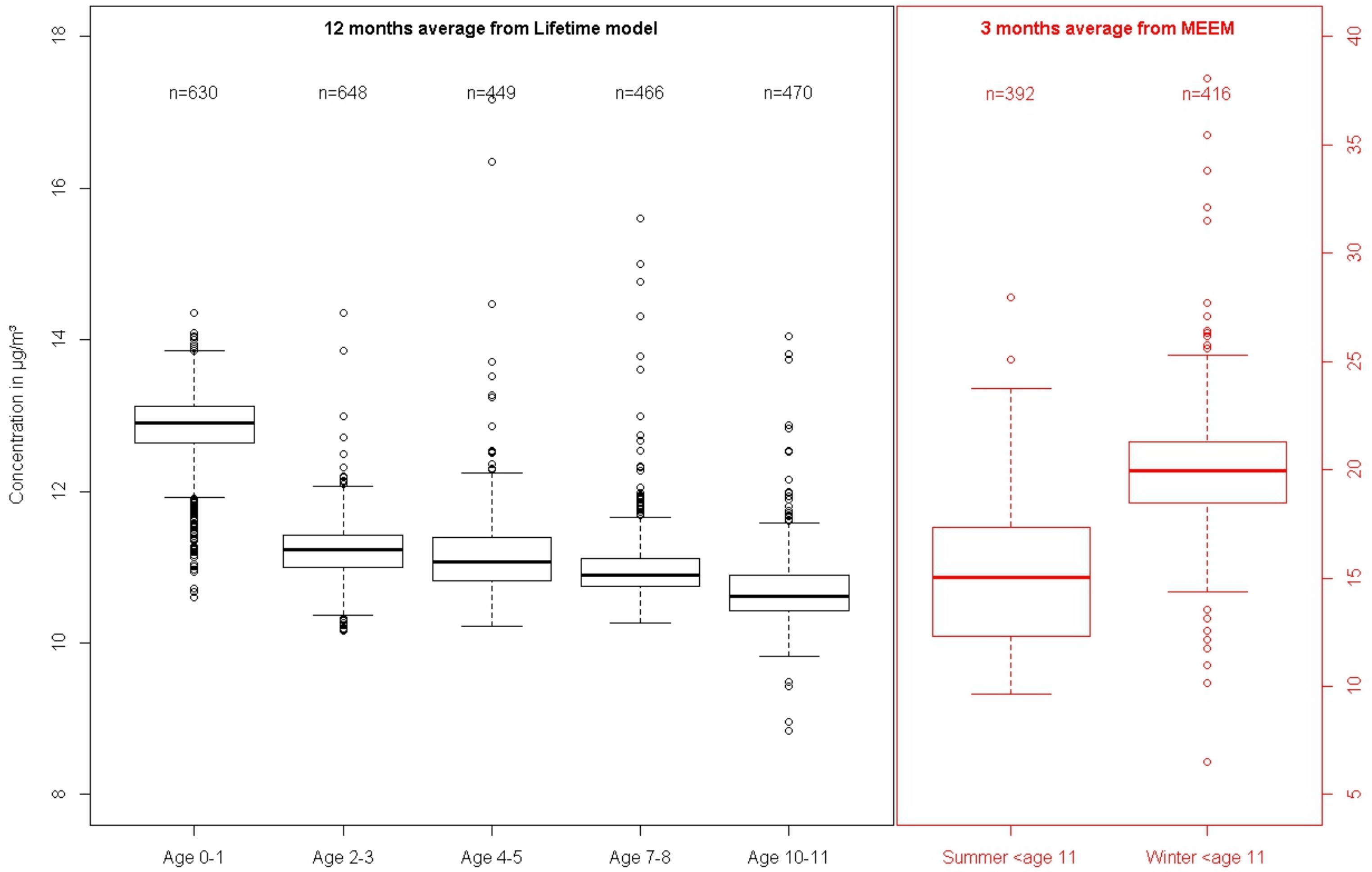

Figure S1: Boxplots of estimated PM_{10} exposures (µg/m³) during the first year of life, one year prior to the review visit [derived from lifetime exposure model] and summer and winter before the age 11 review visit [derived from MEEM] (indicating the median, interquartile range, minimum, maximum and outliers)

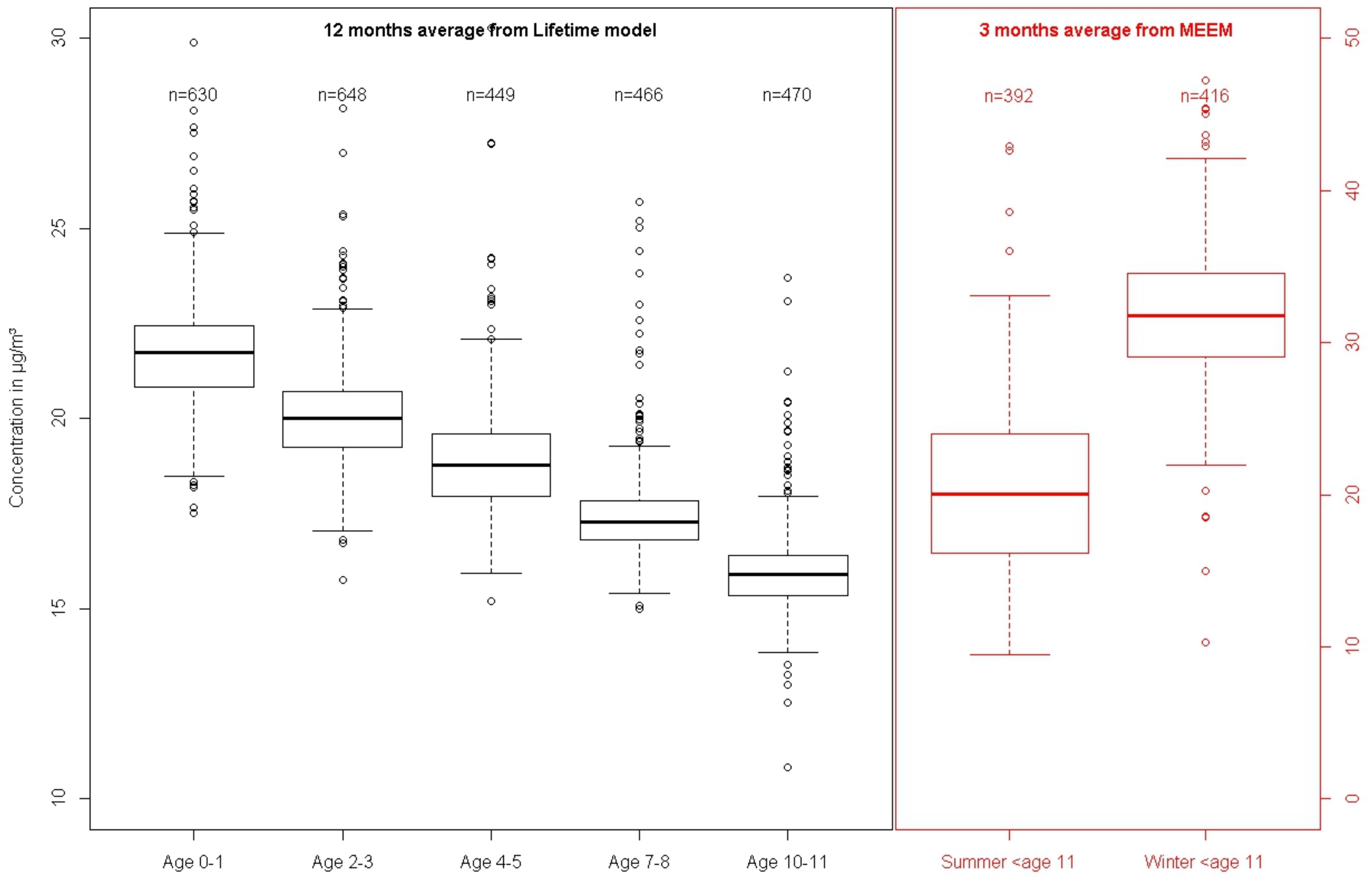

Figure S2: Boxplots of estimated NO_2 exposures (µg/m³) during the first year of life, one year prior to the review visit [derived from lifetime exposure model] and summer and winter before the age 11 review visit [derived from MEEM] (indicating the median, interquartile range, minimum, maximum and outliers)

Table S2: Association between lifetime PM_{10} and NO_2 exposure (in µg/m³) and specific airway resistance at age 3, 5, 8 and 11 years[a]

Exposure window	Outcome Age	PM_{10}			NO_2			
		β[c]	(95% CI)	p	β[c]	(95% CI)	p	n[d]
Age 0-1		-0.048	(-0.090, -0.005)	0.027	-0.016	(-0.031, 0.000)	0.053	339
Age 0-3	3	-0.056	(-0.105, -0.008)	0.023	-0.017	(-0.034, 0.000)	0.054	336
Age 2-3		-0.021	(-0.071, 0.030)	0.422	-0.015	(-0.031, 0.000)	0.056	353
Age 0-1		-0.032	(-0.059, -0.005)	0.019	-0.012	(-0.023, -0.001)	0.025	532
Age 0-5	5	-0.015	(-0.056, 0.027)	0.490	-0.012	(-0.026, 0.002)	0.087	420
Age 4-5		0.021	(-0.005, 0.047)	0.111	0.002	(-0.009, 0.013)	0.710	398
Age 0-1		0.019	(-0.012, 0.051)	0.232	0.006	(-0.006, 0.018)	0.350	513
Age 0-8	8	0.042	(-0.010, 0.093)	0.116	0.013	(-0.007, 0.034)	0.198	313
Age 7-8		0.010	(-0.024, 0.045)	0.548	0.008	(-0.007, 0.023)	0.281	405
Age 0-1		-0.026	(-0.070, 0.018)	0.251	-0.010	(-0.026, 0.007)	0.259	387
Age 0-11	11[b]	0.008	(-0.057, 0.074)	0.807	0.002	(-0.024, 0.027)	0.905	285
Age 10-11		-0.007	(-0.054, 0.040)	0.774	0.002	(-0.020, 0.023)	0.876	352

[a] Adjusted for age, gender, concurrent body mass index, concurrent atopy, concurrent asthma or current wheeze, family history of asthma, hospitalisation during the first two years of life for lower respiratory tract infection, average 3-day background PM_{10} concentration prior to sRaw measurement

[b] Adjusted for Tanner stage [c] β coefficient per 1µg/m³ increase in exposure [d] number of children included in analysis

Table S3: Association between lifetime PM_{10} and NO_2 exposure (in μg/m³) and FEV_1 (% predicted) at age 5, 8 and 11 years[a]

Exposure window	Outcome Age	PM_{10}			NO_2			
		β[c]	(95% CI)	p	β[c]	(95% CI)	p	n[d]
Age 0-1		-2.28	(-4.64, 0.08)	0.058	-0.08	(-0.97, 0.81)	0.86	477
Age 0-5	5	-3.12	(-6.48, 0.23)	0.068	-0.05	(-1.17, 1.07)	0.93	374
Age 4-5		-1.05	(-3.11, 1.02)	0.32	-0.38	(-1.25, 0.49)	0.39	357
Age 0-1		0.36	(-1.52, 2.24)	0.71	0.27	(-0.46, 1.00)	0.47	461
Age 0-8	8	-1.89	(-4.87, 1.08)	0.21	-0.20	(-1.4, 1.01)	0.75	278
Age 7-8		-0.02	(-2.06, 2.01)	0.98	-0.19	(-1.01, 0.71)	0.68	359
Age 0-1		-0.31	(-2.33, 1.71)	0.77	0.04	(-0.74, 0.82)	0.92	413
Age 0-11	11†	-2.07	(-5.12, 0.98)	0.18	-0.84	(-2.03, 0.35)	0.17	302
Age 10-11		-1.13	(-3.36, 1.09)	0.32	-0.83	(-1.79, 0.14)	0.093	373

[a] Adjusted for concurrent atopy, concurrent asthma or current wheeze, hospitalisation for lower respiratory tract infection during the first two years of life, gas cooker in the home (age 8 and 11 only)

[b] Adjusted for Tanner stage

[c] β coefficient per 1μg/m³ increase in exposure

[d] number of children included in analysis

Table S4: Association between lifetime PM_{10} and NO_2 exposure (in µg/m³) and FEV_1 post bronchodilator (% predicted) at age 5 and 11[a]

Exposure window	Outcome Age	PM_{10}			NO_2			
		β[c]	(95% CI)	p	β[c]	(95% CI)	p	n[d]
Age 0-1		-3.00	(-5.29, -0.71)	0.010	-0.91	(-1.77, -0.05)	0.037	439
Age 0-5	5	-4.70	(-7.85, -1.55)	0.004	-0.87	(-1.95, 0.21)	0.11	345
Age 4-5		0.38	(-1.65, 2.42)	0.71	-0.52	(-1.35, 0.30)	0.21	331
Age 0-1		-2.03	(-4.06, 0.01)	0.051	-0.59	(-1.37, 0.20)	0.14	403
Age 0-11	11†	-2.34	(-5.51, 0.84)	0.15	-0.99	(-2.24, 0.25)	0.12	295
Age 10-11		-1.71	(-3.94, 0.53)	0.13	-1.00	(-1.96, -0.03)	0.043	366

[a] Adjusted for concurrent atopy, concurrent asthma or current wheeze, hospitalisation for lower respiratory tract infection during the first two years of life, gas cooker in the home (age 11 only)
[b] Adjusted for Tanner stage
[c] β coefficient per 1µg/m³ increase in exposure
[d] number of children included in analysis
